# Community-driven mental health priorities for immigrant youth in Alberta

**DOI:** 10.3389/frhs.2025.1658656

**Published:** 2025-11-03

**Authors:** Syeda Farwa Naqvi, Mungunzul Amarbayan, Gina Dimitropoulos, Jennifer Zwicker, Maria Jose Santana

**Affiliations:** ^1^ Cumming School of Medicine, University of Calgary, Calgary, AB, Canada; ^2^ Medical College of Wisconsin, Milwaukee, WI, United States; ^3^ Faculty of Social Work, University of Calgary, Calgary, AB, Canada; ^4^ School of Public Policy, University of Calgary, Calgary, AB, Canada

**Keywords:** James Lind Alliance priority partnership, immigrant youth, mental health, nominal group technique (NGT), community engagement

## Abstract

**Background:**

Immigrant youth population is more susceptible to poor mental and overall health due to environmental factors, such as higher risks of poverty, trauma, displacement, and settlement period, learning a new language, adapting to a new culture, and a lack or loss of social supports. The overall goal of this project was to identify the research priorities of immigrant youth with lived experience of mental health concerns to guide research in mental health and inform health policy in a partnership with community organizations across Alberta, Canada.

**Methods:**

This patient-oriented research was designed based on the James Lind Alliance Priority Setting Partnership five steps: (1) creating a steering committee; (2) gathering uncertainties (questions which cannot be answered by existing research); (3) refining uncertainties through steering committee; (4) prioritization with immigrant youth via focus groups and with stakeholder involved in the care of immigrant youth through a nominal group technique; and (5) finalizing priority setting, report and dissemination. A steering committee was created with immigrant youth who self-identified with lived experience of mental health issues, leaders from immigrant communities (aged 18–25), researchers, non-profit organization leaders, and healthcare or community service providers. The electronic survey was distributed in rural, remote, suburban, and urban settings to recruit self-identified immigrant (“someone who has permanently located in a country other than their place of home origin”) youth between the ages of 15 and 25 residing in Alberta, Canada.

**Results:**

Based on 148 responses from immigrant youth with a mental health concern, 25 uncertainties were refined. The top five priorities were chosen at the focus groups and NGT. Youth prioritized uncertainties related to them and their communities, while key informants emphasized higher-level uncertainties (resources, institutional barriers). Both prioritized community roles in reducing stigma, schools’ role in addressing mental health, and the impact of COVID-related isolation.

**Conclusions:**

This study underscores the need for policies that support the tailoring of mental health services to the individual needs of immigrant youth. The findings from this study affirm that immigrant youth recognize mental health as not linear or universal; they seek to support each other and advocate for systemic changes that increase literacy and access to care.

## 1 Introduction

Canada has one of the highest rates of immigration in the world resulting in 23% of the population being immigrants according to the census in 2021, with Alberta being one of the fastest-growing provinces mainly due to international migration (1, 2). One in 10 recent immigrants in Canada are youth or young adult aged 15–24 (10.9%), with this group projected to make up between 39% and 49% of the total population of children aged 15 years and under by 2036 (3–5).

Environmental factors that cause higher susceptibility of immigrant youth population to poor mental and overall health including higher risks of poverty, trauma, displacement, and settlement period, learning a new language, adapting to a new culture, and a lack or loss of social supports, often lead to bullying, racial, ethnic, and cultural discrimination (5–7). Time since migration is another important factor that plays a role in the mental health of the immigrant population. Salami et al. (8) studied mental health problems among immigrant and non-immigrant people in Canada and identified that overall, there were no differences between the groups in the first years of living in Canada which is known as “healthy immigrant effect.” When adjusting for “time from immigration,” people who immigrated within 5 years had better self-reported mental health than immigrants who migrated 10 years before (8). Approximately 4.6 million Canadians predominantly speak a language other than English or French at home which represents 12.7% of the population. In Alberta, this number reached 13% in 2021 (9). Language barriers may exacerbate negative mental states in immigrant youth such as anxiety and depersonalized interactions, delay access to mental health services due to limited communication in English and French, and increase feelings of social alienation as immigrant youth struggle to connect meaningfully with others in their new environment (10, 11). The culmination of these and many other factors can result in serious and even fatal consequences, as seen in the tragic death of 9-year-old Syrian refugee, Amal Alshteiwi, in Calgary in 2019 who took her life after being bullied at school for 6 months unrecognized, unchecked, and unreported because, according to family friends, much of it occurred in Arabic and was not understood by English-speaking staff (12).

In response to this terrible accident and to the growing needs of immigrant refugee youth mental health, the local immigrant and refugee organizations joined forces to establish the United Voices Committee in Alberta, Canada (13). The United Voices is a local coalition of approximately 25 immigrant-related community organizations that aims to reduce stigma and promote mental health awareness among immigrant youth. In partnership with the Mental Health Commission of Canada, the committee organized the inaugural United Voices Summit in October 2019, which brought together 200 immigrant youth from Calgary who highlighted the need to address the existing gaps related to mental health (13).

This project was ignited by feedback from immigrant youth described above prompting a strong partnership with the United Voices Committee and other community organizations across the province, which supported the engagement throughout the study.

The overall goal of this study was to identify the research priorities of immigrant youth with lived experience of mental health concerns to guide research in mental health and inform health policy.

## 2 Materials and methods

This patient-oriented research study follows the James Lind Alliance (JLA) process to assess the priorities of immigrant youth (14). In this study, the term “immigrant” refers to “someone who has permanently located in a country other than their place of home origin” (15). The University of Calgary Conjoint Health Ethics Board approved this study (REB19-1784).

### 2.1 James Lind Alliance Priority Setting Partnership

The JLA is an organization that developed a standardized Priority Setting Partnership (PSP) project guidelines for joint decision-making between patients, researchers, and/or providers to guide research (14).

**Figure 1:**
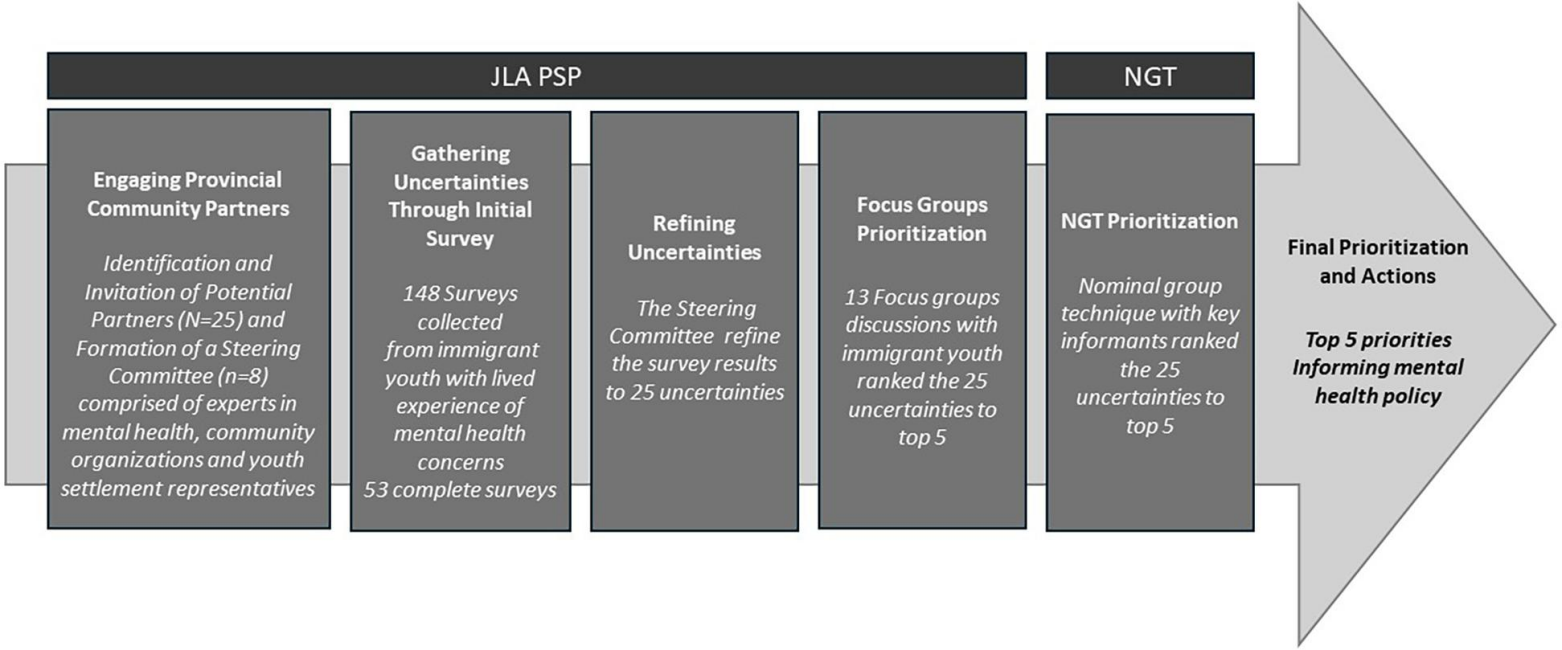
Flowchart depicting the project at a glance.

The JLA process was chosen for this study as it is designed for priority setting in health research by bringing together patients, community, and clinicians to identify and prioritize uncertainties (questions that cannot be answered by existing research) that are important to all stakeholder groups. To support this study, a strong partnership with community organizations was established. Figure 1 depicts the project briefly.

The JLA PSP created a stepwise framework for patients and healthcare professionals to identify healthcare priorities for all parties that could be addressed and enhanced through research (14). This study was designed based on the JLA PSP five steps: (1) engaging provincial community partners creating a steering committee; (2) gathering uncertainties; (3) refining uncertainties through steering committee; (4) prioritization with immigrant youth via focus groups and with stakeholder involved in the care of immigrant youth through a nominal group technique (NGT); and (5) finalizing priority setting, report and dissemination (see Figure 1).

#### 2.1.1 Engaging provincial community partners

During this step, the focus was to identify and invite partners to create a steering committee that will guide us throughout the study. Steering committee members were recruited through connections with the Alberta Strategy for Patient-Oriented Research Support Unit (AbSPORU) (16). AbSPORU’s role is to support patient and community engagement in Alberta. We requested community engagement support from AbSPORU for this research project. AbSPORU connected us with non-profit organizations including United Voices Committee (a network of 25 immigrant-related community organizations) (5), Immigrant Services Calgary, and a representative from the Mental Health Commission of Canada in Alberta. A steering committee was created with individuals working in immigrant services interested in mental health who had the capacity to contribute to this project as volunteers. The committee included eight people who met monthly and were engaged in every step of the study, from developing survey questions and their dissemination to evaluation of results and prioritization approaches including focus groups and NGT.

#### 2.1.2 Gathering uncertainties (initial survey dissemination/data collection)

Self-identified immigrant youth with lived experience of mental health issues between the ages of 15 and 25 residing in Alberta, Canada, were recruited to participate in the survey and focus groups.

The electronic survey was co-created by the University of Calgary research team in collaboration with the steering committee. The first part of the survey included questions about demographic background, languages spoken at home, immigration history, and previous lived experience with mental health concerns. The second part of the survey was a text-free field for young people to list any questions they had about mental health care broadly. This survey was hosted through the University of Calgary's licensed Qualtrics survey platform. The completion time was 5–10 min, and the mode of completion was electronic via phone, tablet, or computer.

The survey distribution was supported by the steering committee through their networks that received email invitations with a link to direct the participants to the survey. Survey distribution was also supported by the AbSPORU from January to February 2020. The survey was distributed in both rural, remote, suburban, and urban settings in Alberta, Canada. Consent was obtained from the youth to be contacted for future participation in focus groups.

#### 2.1.3 Refining uncertainties (researchable questions)

After obtaining survey results from youth responders, the steering committee assisted in categorizing the various mental health-related questions from the second part of the survey into uncertainties. This categorization resulted in 25 uncertainties that were presented to focus groups and NGT participants for prioritization at the next stages of the study.

#### 2.1.4 Prioritization

The prioritization phase consisted of presenting the survey findings, 25 uncertainties, to immigrant youth participants in focus groups and to key informants via NGT.

##### 2.1.4.1 Focus groups

A total of 13 focus groups were conducted across the province of Alberta including rural and remote areas proportional to the density of the geographical area. Twelve of them were held virtually on the Zoom virtual communication platform which was pivotal to remaining compliant with the government-issued COVID-19 pandemic restrictions. Furthermore, the virtual focus groups allowed participants to share their thoughts through the chat box. To include diverse immigrant youth, focus groups were offered in English, French, Arabic, Mandarin, and Tagalog. Most focus groups were conducted in English except for one held in French in the rural city of Brooks, Alberta, Canada, which was conducted in person with the francophone rural community due to the lack of resources and poor WIFI connection. Following the COVID-19 restrictions, this focus group was conducted in a community center with recent young African francophone immigrants.

Previous survey participants who consented to be contacted to join the focus groups were invited to participate. At the start of the focus groups, participants provided oral consent. Participants were presented with the overall study and asked for oral consent that was recorded. These participants received a $25 gift card and a certificate recognizing two hours of their participation.

During the focus groups, participants were introduced to the research project and 25 uncertainties from the initial survey as a slide show. These slides were accessible to the participants on a Google Drive, making them available throughout the session. Participants were given time to review the questions. They were asked to rank their top five uncertainties and sent them through the chat feature of the Zoom platform to the focus group facilitator. Each participant was allowed time and space to share their perspective, discussing their choices while ranking them. The output at the end of each focus group was the top priorities. Researchers compiled all these priorities from each of the focus groups and counted them identifying the most frequent ones. The results are described in the next section of the paper.

##### 2.1.4.2 Nominal group technique

Nominal group technique is a method that allows individuals to think about a given topic in complete silence for a finite time and then write down contributions for discussion without group influence (17, 18). The NGT method was an appropriate choice because it enabled us to discuss sensitive and controversial topics such as immigrant and refugee youth mental health and then reach an agreement that is satisfactory to the group as a whole (17, 18).

The NGT included five steps: (1) *silent generation* at which each participant silently generated and wrote down their ideas related to the specific topic; (2) *sharing their ideas* one at the time without discussion; (3) *idea clarification* to ensure that everyone understand the topic; (4) *preliminary round of ranking* privately; and (5) *final ranking* when the group discussed the prioritized uncertainties from the list of 25 uncertainties.

The NGT participants included key informants with relevant experience in immigrant youth mental health. The participants were recruited through the networking efforts of the steering committee, from provincial organizations working in the immigrant youth sector of Alberta by using a convenience sampling approach. Participants included local Albertan immigrant community leaders and academics with experience in immigrant youth mental health research.

These participants received the 25 uncertainties from the initial survey and the top priorities from the earlier JLA focus groups with immigrant youth. Participants wrote down their rankings of the top five uncertainties. During the NGT process, the participants had the opportunity to privately write their personal ranking and opinions which the researchers could read and consider in the discussions to accommodate those with opposing views and thoughts. The group had a discussion based on the independent top five rankings. The final output of this ranking exercise was the NGT's top priorities.

#### 2.1.5 Final prioritization

The final stage aimed to summarize findings and plan the dissemination strategies with the overall aim of informing mental health policy. This paper is one aspect of the research dissemination stage.

## 3 Results

### 3.1 Collecting uncertainties: survey results

#### 3.1.1 Sociodemographic characteristics

Participants were younger than 24 years old, 71% were female, and 29% were born in Canada, with 26% speaking only English at home. Additionally, 21% lived in rural areas, and 79% lived in urban areas and surrounding suburban zones, with 53% living in Calgary. Most of them (45%) were experiencing mental health problems at the time of completing the survey, 26% experienced mental health issues in the past, and 29% had experience taking care of others living with mental health issues.

#### 3.1.2 Data collection

Between January and February 2020, a total of 148 surveys were received. Seventy-two surveys were excluded, three youth didn't sign the consent to participate, and 69 surveys had no uncertainties. Out of the 76 surveys with responses, 53 of them provided complete responses. These 53 uncertainties were refined; for instance, stigma-related uncertainties were framed in different ways and refined into the resulting priority 18. At the end of this phase, the final 25 uncertainties were presented to participants at the focus groups and at the NGT. See Supplementary Table S1.

##### 3.1.2.1 Focus groups with immigrant youth

Sixty-one immigrant youth who participated in the survey participated in the 13 focus groups. Most of the respondents were born outside of Canada (74.2%), and 64% self-identified as having mental health challenges before or at the time of the study. Supplementary Table S2 lists the demographic characteristics of focus group participants.

There were 41% male, 52% female, and 6% non-binary/gender fluid participants. The mean age of participants was 16 years old. The diverse ethnic heritage of the sample included Black (48%), Filipino (20%), Chinese (15%), Middle Eastern (10%), White (7%), and Southeast Asian (2%). In terms of country of birth, the majority were born in Canada (28%), followed by the Philippines (20%), Syria (8%), Sudan (8%), and the United States (6%), with the remaining 29% representing various other countries.

Most of the participants were fluent in English (34%) except for the African francophone group (16%). One participant did not share ethnic heritage, country of birth, or languages spoken. The rest 29 participants’ languages included Tagalog (15%), Arabic (12%), Mandarin/Cantonese (10%), Tigrinya (5%), and South Sudanese languages (3%), and a minority spoke Bisaya, Sinhala, Kurdish, and Tadaksahak.

Thirteen focus groups were conducted including 61 participants from various cities, rural and remote towns in Alberta, Canada. The number of focus groups per geographical area was aligned with population density resulting in six in Calgary, three in Edmonton, three in Southern Alberta, and one in Northern Alberta. Out of the 61 respondents, 49% had current experiences with mental health concerns, while 31% had mental health problems in the past and 20% rest were caregivers.

Uncertainties collected from each of the focus groups were mapped to each other to identify the common ones across rankings within the top five. These final priorities were ranked by focus groups four or more times. This was conducted by three researchers in Excel, counting the frequency (see Supplementary Table S3).

Out of the 25 uncertainties, seven of them informed the top five priorities for immigrant youth.

The following uncertainties were ranked the highest priority, in descending order:

• 13) How can mental health be improved?
• 10) How does mental illness affect education, employment, and job opportunities?
• 18) How can communities reduce stigma to help youth access care?
• 14) What factors lead to or worsen mental health challenges?
• 22) How can I support someone struggling with mental health?
• 24) What is the impact of isolation on youth during the COVID-19 pandemic?
• 16) How can schools better address mental health challenges?

The last three were a tie and included based on the recommendations of the steering committee. Furthermore, the steering committee emphasized the importance of including priority number 24 because it was contextual, as the focus groups were conducted during the COVID-19 pandemic.

##### 3.1.2.2 NGT with stakeholders in the immigrant youth and mental health field

The NGT was held with 18 key informants with experience in immigrant youth and mental health. Participants included a diverse group of individuals who immigrated from different countries, had experience in working with immigrant youth in community organizations, and were experts in the field of mental health research, social work, and health policy.

The group represented a wide demographic range, including researchers, and experienced practitioners working in immigrant-serving organizations [e.g., Benevolent Chinese Society (19) in Edmonton and Immigrant Services Calgary (20)], women's associations [e.g., Calgary Immigrant Women Association (21)], and youth advisory councils in non-profit organizations [e.g., United Voices (22)].

Participant's contributions were informed by both lived experiences of migration from countries including countries such as Mexico, Ukraine, Russia, Israel, and the Philippines and professional expertise in mental health and social services. Collectively, they work together as key informants, bridging the experiences of migrant youth in Alberta with the available community resources and institutional supports. Each participant offered a distinct standpoint, underscoring structural barriers, cultural dynamics, and the imperative for more responsive and collaborative approaches to addressing immigrant youth mental health.

The results of their ranking included the following prioritized uncertainties ranked in descending order:

• 3) What resources exist specifically for immigrant and newcomer youth, ethnocultural youth, and refugees in Alberta?
• 8) What are individual factors that might prevent someone from reaching out or seeking help for mental health issues?
• 16) How can schools better address mental health challenges?
• 18) How can communities reduce stigma to help youth access care?
• 24) What is the impact of isolation on youth during the COVID-19 pandemic?
• 2) What are the structural barriers to receiving care, and how can we improve access?

Priority numbers 24 and 2 were a tie. The steering committee recommended keeping it.

During the NGT discussions, the key informant participants recognized the disintegrated systems and emphasized the importance of collaboration between the government, healthcare system, and community organizations to build support networks for immigrant youth. Furthermore, NGT participants highlighted the lack of consistent and comprehensive guidelines regarding culturally competent mental health care and the need to develop strategies to address cultural competence.

Supplementary Table S4 shows a comparison of the top priorities between youth participants in focus groups and key informant participants in the NGT. Although the purpose was to refine the prioritization to the top five ones, three of the uncertainties, i.e., 22, 24, and 16, were ranked equally by the youth in different focus groups; and two of the uncertainties discussed by the key informants, i.e., 24 and 2, were ranked equally by the NGT participants.

It's important to highlight that while the youth prioritized uncertainties related to them personally and their communities, the key informants highlighted the uncertainties at a higher level including resources and institutional barriers. However, both groups prioritized the role of communities in reducing stigma to help access to mental health care, the impact of isolation due to COVID-19 on youth mental health considering that focus groups were held at the height of government-issued COVID-19 pandemic restrictions as well as the role of schools in helping to address mental health. Participant C stated the following:

If you're going to look at the school administration level that sometimes the schools get overwhelmed with the number of third-party organizations support too… school plays an important role to really knowing what their students need so that they know which service provide they can really tap into. But at the same time schools maybe they are overwhelmed with the amount of the demands of the students [and] that the… supplies they are receiving isn't really fitting into their students need.

## 4 Discussion

This study aimed to identify the research priorities of immigrant youth with lived experience of mental health concerns to guide research, inform health policy, and learning health systems. Using the James Lind Alliance Priority Setting Partnership (JLA PSP) approach, we investigated the needs and high-priority focus areas for immigrant youth mental health in Alberta through surveys, focus groups with immigrant youth, and a nominal group technique with key informants. The survey identified 25 uncertainties relevant to immigrant youth, which after being prioritized resulted in the top five priorities centered around improving mental health, understanding external influences, and enhancing resources and supports. The findings from this study show that immigrant youth hope to see mental health services and supports tailored to the personal needs of immigrant youth living with mental health concerns.

Mental health is experienced and discussed differently across diverse ethnic groups, shaped by unique histories, cultural norms, and societal contexts (3). Recognizing this, this study found that youth in urban and rural settings prioritized questions exploring how mental health can be improved (priority 13) and how it affects education and employment (priority 10). These priorities highlight a holistic understanding of mental health's role in achieving life goals and navigating cultural and systemic challenges in Canada (23, 24). Interesting to learn that in the United States, the Health Equity and Accountability Act of 2020 supports culturally responsive mental health initiatives for underrepresented Black youth (25). However, no equivalent policy exists in Alberta, Canada.

Priority number 10, which questions how mental health affects education, employment, and job opportunities, underscores youth's awareness of the critical link between mental health and future opportunities. Existing research also highlights that navigating visas, education, and employment in a new cultural context increases stress and risk of mental health concerns (26). Cultural stigmas and taboos about mental illness can further compound these risks (27–30).

Youth, especially in urban settings, also recognized the reciprocal relationship between mental health and external circumstances that was reflected in the equal ranking of priority 14 (what factors lead to or worsen mental health challenges?). Both focus groups and NGT participants prioritized uncertainty 18 (how can communities reduce stigma to help youth access care?). These priorities address gaps in mental health literacy and the stigma in their own communities about living with mental health concerns. Stigma in some cultures may frame mental health challenges as weakness or moral failing, and seeking help may be viewed as culturally unacceptable (5). Participant B added to the idea of stigma within immigrant communities creating barriers by saying that someone reaching out to get the help they need maybe met with deterring comments about mental health such as “pull yourself up with your socks and should not be taking any anti-depressants, what's wrong with you…you're weak and so on” and that “different cultures have different views.” Language barriers can intensify stigma, making discussions about mental health even more difficult. Community education about signs of mental health concerns, especially involving community leaders, can reduce stigma, normalize conversations about mental health, and increase acceptability of mental health resources. Improving mental health literacy and access to services can, in turn, enhance outcomes, which also relate to the final priorities ranked by immigrant youth (31–33).

The idea that immigrant youth are inherently resilient because of cultural values emphasizing family, community, and perseverance is a common misconception (29, 30, 34). While family and community supports can foster resilience (28), they can also mask vulnerability; youth who experience discrimination, marginalization, or a lack of support may face heightened mental health risks (30). This tension between stigma and resilience was highlighted by the participants in the focus groups. Specifically, regarding how resilience varies based on family and community context. Immigrant youth emphasized the need to understand the issue of the often false assumption of immigrant youth's mental health resilience by healthcare system leaders and policymakers.

Priority 22 reflected the youth interest in peer support especially in the rural areas where mental health support services are not widely available, which can be tied to priority 16 about school-based mental health strategies and priority 24 that touches the profound impact of the COVID-19 pandemic. School serves as a critical space for socialization and support, especially when the home environment carries stigma (35). The COVID-19 pandemic disrupted this support system, leading to worsened outcomes (35–38). While telehealth improved access for some, it also excluded youth in low-income and rural areas without reliable technology (39). An example was the reason to conduct the in-person focus groups in the rural area of Brooks, Alberta.

An indication of youths’ awareness that mental health is dynamic, evolving with context and events, was the prioritization of uncertainty 24 about the impact of isolation during the COVID-19 pandemic. This aligns with research showing significant increase in a number of youths using mental health care (from 15.6% in 2018/2019 to 18.8% in 2021/2022) which was affected by an immediate drop in a use of mental health care in April 2020 when COVID-19 restrictions were introduced and followed by steady increase during next two years which support the need for tailored interventions (35–38). Notably, participants in focus groups and NGT identified pandemic-related mental health impacts as a priority, underscoring their broad significance.

In contrast to youths’ emphasis on personal and community aspects of mental health, key informant participants in NGT prioritized upstream and systemic strategies to enhance services and resources for immigrant youth, which is consistent with literature emphasizing the need for structural change (29). In the NGT group, Participant F pointed out that often, “it is not the lack of mental health resources but rather it is because they are lacking in how they've been structurally created or in how they've been set up to facilitate these services,” in reference to a niche population with niche concerns.

Ungar's (40) concept of cultural relativism supports this approach, advocating those cultural practices and beliefs be understood within their context rather than judged by external norms. This perspective is essential for reducing stigma and avoiding misdiagnosis. For instance, research shows Black and Spanish adolescents are more often misdiagnosed with attention deficit hyperactivity disorder compared with their Caucasian peers, partly due to providers’ limited cultural understanding (41). Applying cultural relativism can help professionals identify culturally rooted determinants of mental health and design interventions that leverage community strengths rather than override them (40). Cultural humility training can further improve care by encouraging self-reflection, recognizing biases, and addressing power imbalances (29).

The findings from this study affirm that immigrant youth recognize mental health as not linear or universal; they seek to support each other and advocate for systemic changes that increase literacy and access to care, while emphasizing tailoring services and treatments to the individual needs of immigrant youth.

The urgency and importance of investing in and developing a comprehensive mental healthcare system for youth in Canada have been an ongoing discussion for many years. Large-scale initiatives, such as ACCESS Open Minds (ACCESS-OM), proved that involving youths, families, Indigenous communities, and healthcare partners, researchers, and decision-makers in mental health service and care design enhances timely access and effectiveness of mental health support (42, 43). However, addressing immigrant youth mental health remains complex due to the intersecting cultural, systemic, and individual factors involved. This study helps to fill knowledge gaps by centering perspectives of immigrant youth with lived experience of mental health concerns on what matters most to them. These priorities can guide program development and policy decisions to better meet the mental health needs of immigrant youth, to understand the influence of cultural views and the Canadian context on their well-being. Given its foundational nature, this study acts as a baseline for other researchers to build on it and further investigate the intricacies of how each of the identified priorities can be maneuvered and harnessed into meaningful and dynamic impacts that can stand the test of time. Branching this study off to explore the experiential differences between youth from different demographics within Alberta (rural, urban, women's specific issues, physical disabilities with acquired mental health concerns, immigrant, and cultural implications, and/or LGBTQ) would help us gain a better understanding of the specific mental health needs and approaches for various cohorts and demographics.

## 5 Strengths and limitations

The partnership established with non-profit organizations and key informant experts in mental health and immigrant health leading to the creation of the steering committee was one of the foundational strengths of this project. Without their support in recruiting participants, this study across the large geographical extension of Alberta would not have been possible.

The inclusion of immigrant youth from urban, rural, and remote areas of Alberta facilitated gathering uncertainties from a diverse sample of immigrant youth. Furthermore, the virtual focus groups diminished the risk of potential barriers to in-person focus groups by saving time for travelling. On the other hand, an in-person focus group was needed to overcome access to WIFI in remote rural areas.

A limitation of the virtual focus groups was that, for participants who had their cameras turned off, we were unable to observe facial expressions and gestures that might have offered valuable non-verbal insights. However, providing flexibility in online participation allowed those who felt less comfortable speaking aloud to share their perspectives through the chat function instead.

This study underscores the critical role of patient-oriented research in addressing the unique mental health challenges faced by immigrant youth in Alberta. By engaging youth as partners rather than subjects, these findings foster culturally responsive insights that can inform learning health systems to adapt in real-time to evolving community needs. Incorporating lived experiences from youth enables the identification of structural barriers, such as language, stigma, and limited resource knowledge and awareness, that disproportionately affect immigrant youth. Embedding these insights into Alberta's learning health systems presents an opportunity to co-develop targeted interventions, improve care pathways, and build trust with historically underserved populations. This work illustrates how meaningful youth engagement could contribute to a more inclusive, responsive, and equitable learning health system.

## 6 Conclusion

This study aimed to explore what immigrant youth themselves identify as important regarding mental health challenges and service delivery. The findings from focus groups reveal the unique mental health priorities of immigrant youth, which could inform mental health policy and support learning health systems in personalizing care. Although policies and programs have been established to address youth mental health concerns in Canada, existing research often treats youth as a single, uniform demographic or portrays immigrants as a homogeneous group. Consequently, research specifically examining the distinct mental health needs of immigrant youth remains limited. This study underscores the need for policies that support the tailoring of mental health services. Furthermore, these priorities should guide future research on mental health for immigrant youth from different communities in Canada and beyond.

## Data Availability

The raw data supporting the conclusions of this article will be made available by the authors, without undue reservation.
