# Supplementary material for: Community-driven mental health priorities for immigrant youth in Alberta
Source: Front Health Serv. 2025 Nov 3;5:1658656. doi: 10.3389/frhs.2025.1658656 (PMC12620385; doi:10.3389/frhs.2025.1658656)
Supplement: Supplementary file 1 [file Datasheet1.pdf]

Table 1. Characteristics of focus groups participants N=61*

Gender	
Male	25 (41)
Female	32 (52)
Non-Binary/ Gender-fluid	4 (6)
Race/Ethnicity	
Black	29 (48)
Filipino	12 (20)
Chinese	9 (15)
Middle Eastern	6 (10)
White	4 (7)
Southeast Asian	1 (2)
Country of Birth	
Canada	17 (28)
Philippines	12 (20)
Syria	5 (8)
Sudan	4 (7)
USA	3 (5)
Britain	2 (3)
Egypt	2 (3)
Uganda	2 (3)
Mexico	1 (2)
Mali	1 (2)
Ethiopia	1 (2)
Rwanda	1 (2)
Nigeria	1 (2)
Sri Lanka	1 (2)
Ghana	1 (2)
Language Spoken	
English	21 (34)
French	10 (16)
Tagalog	9 (15)
Arabic	7 (12)
Mandarin/Cantonese	6 (10)
Tigrinya	3 (5)
South Sudan: Kuru, Madi, Shilluk	2 (3)
Others: Kurdish, Tadaksahak, Bisaya, Kinyarwanda	5(8)

Notes: * missing data from one participant
