# Supplementary material for: Community-driven mental health priorities for immigrant youth in Alberta
Source: Front Health Serv. 2025 Nov 3;5:1658656. doi: 10.3389/frhs.2025.1658656 (PMC12620385; doi:10.3389/frhs.2025.1658656)
Supplement: Supplementary file 2 [file Datasheet2.pdf]

Table 2. Twenty-five uncertainties (questions) presented at Focus Groups for prioritization.

#	Uncertainty (question)
1	How accessible is therapy or medical help for youth in Alberta?
2	What are the structural barriers to receiving care and how can we improve access?
3	What resources exist specifically for immigrants and newcomer youth, ethnocultural youth and refugee in Alberta?
4	Is there a difference in the number of resources available to immigrant and newcomer youth compared to other youth?
5	Are there cultural differences in how individuals and communities can approach or seek treatment for mental health issues?
6	What is the proportion of youth living with mental health concerns who have not sought help?
7	Is there an under representation of immigrant and refugee youth in those seeking mental health treatment?
8	What are the individual factors? That may prevent someone from reaching out or seeking help for mental health issues?
9	Does social media impact the mental health of immigrants and newcomers compared to other youth?
10	How does mental health affect education, employment and job opportunities?
11	What is long term impact of mental health on the health and identity of youth from immigrant and newcomers' groups?
12	What is the biological mechanism behind mental health?
13	How can mental health be improved?
14	What factors lead to or worsen mental health challenges?

15	How prevalent are mental health conditions and symptoms (anxiety, depression) amongst youth?
16	How can school better address mental health challenges?
17	What are the incorrect assumptions that people make about mental illness?
18	How can communities reduce stigma to help youth access care?
19	How do ethnocultural communities in Alberta view mental health?
20	How do we address mental health in culturally and spiritually relevant ways?
21	What is the role of spirituality in mental health?
22	How can I support struggling with mental health?
23	What are the experiences of youth living with mental health challenges during COVID-19 pandemic?
24	What is the impact of isolation on youth during the COVID-19 pandemic?
25	What are the protective factors that make some youth more resilient than others to mental health challenges?
