# Supplementary material for: Community-driven mental health priorities for immigrant youth in Alberta
Source: Front Health Serv. 2025 Nov 3;5:1658656. doi: 10.3389/frhs.2025.1658656 (PMC12620385; doi:10.3389/frhs.2025.1658656)
Supplement: Supplementary file 3 [file Datasheet3.pdf]

Table 3. Top priorities selected by immigrant youth and key informants

Immigrant Youth, N = 61	Key Informants, N =18
13. How can mental health be improved?	3. What resources exist specifically for immigrant and newcomer youth, ethnocultural youth, and refugees in Alberta?
10. How does mental illness affect education, employment, and job opportunities?	8. What are individual factors that might prevent someone from reaching out or seeking help for mental health issues?
18. How can communities reduce stigma to help youth access care?	16. How can schools better address mental health challenges?
14. What factors lead to or worsen mental health challenges?	18. How can communities reduce stigma to help youth access care?
22. How can I support someone struggling with mental health?	24. What is the impact of isolation on youth during the COVID-19 pandemic?
24. What is the impact of isolation on youth during the COVID-19 pandemic?	2. What are the structural barriers to receiving care, and how can we improve access?
16. How can schools better address mental health challenges?	

Note: Although the purpose was to refine the prioritization to the top five ones, three of the uncertainties 22, 24 and 16 were ranked equally by the youth in different focus groups; and two of the uncertainties discussed by the key informants, 24 and 2 were ranked equally by the NGT participants.
